# Protection against Tuberculosis in Eurasian Wild Boar Vaccinated with Heat-Inactivated *Mycobacterium bovis*

**DOI:** 10.1371/journal.pone.0024905

**Published:** 2011-09-14

**Authors:** Joseba M. Garrido, Iker A. Sevilla, Beatriz Beltrán-Beck, Esmeralda Minguijón, Cristina Ballesteros, Ruth C. Galindo, Mariana Boadella, Konstantin P. Lyashchenko, Beatriz Romero, Maria Victoria Geijo, Francisco Ruiz-Fons, Alicia Aranaz, Ramón A. Juste, Joaquín Vicente, José de la Fuente, Christian Gortázar

**Affiliations:** 1 NEIKER-Tecnalia, Animal Health Department, Derio, Bizkaia, Spain; 2 Instituto de Investigación en Recursos Cinegéticos IREC (CSIC – UCLM – JCCM), Ciudad Real, Spain; 3 Chembio Diagnostic Systems Inc., Medford, New York, United States of America; 4 Centro de Vigilancia Sanitaria Veterinaria (VISAVET), Facultad de Veterinaria, Universidad Complutense de Madrid, Madrid, Spain; 5 Department of Veterinary Pathobiology, Center for Veterinary Health Sciences, Oklahoma State University, Stillwater, Oklahoma, United States of America; University of Palermo, Italy

## Abstract

Tuberculosis (TB) caused by *Mycobacterium bovis* and closely related members of the *Mycobacterium tuberculosis complex* continues to affect humans and animals worldwide and its control requires vaccination of wildlife reservoir species such as Eurasian wild boar (*Sus scrofa*). Vaccination efforts for TB control in wildlife have been based primarily on oral live BCG formulations. However, this is the first report of the use of oral inactivated vaccines for controlling TB in wildlife. In this study, four groups of 5 wild boar each were vaccinated with inactivated *M. bovis* by the oral and intramuscular routes, vaccinated with oral BCG or left unvaccinated as controls. All groups were later challenged with a field strain of *M. bovis*. The results of the IFN-gamma response, serum antibody levels, *M. bovis* culture, TB lesion scores, and the expression of C3 and MUT genes were compared between these four groups. The results suggested that vaccination with heat-inactivated *M. bovis* or BCG protect wild boar from TB. These results also encouraged testing combinations of BCG and inactivated *M. bovis* to vaccinate wild boar against TB. Vaccine formulations using heat-inactivated *M. bovis* for TB control in wildlife would have the advantage of being environmentally safe and more stable under field conditions when compared to live BCG vaccines. The antibody response and MUT expression levels can help differentiating between vaccinated and infected wild boar and as correlates of protective response in vaccinated animals. These results suggest that vaccine studies in free-living wild boar are now possible to reveal the full potential of protecting against TB using oral *M. bovis* inactivated and BCG vaccines.

## Introduction

Tuberculosis (TB) caused by *Mycobacterium bovis* and closely related members of the *Mycobacterium tuberculosis complex* (MTBC) is a chronic disease that causes huge losses to the cattle industry [1], has consequences on wildlife management and conservation [2], and is a zoonosis affecting millions of people, mainly in developing countries where it causes 10% of human TB cases [3]. The increase in the incidence of bovine TB in some developed countries is thought to be due, at least in part, to wildlife reservoirs of *M. bovis* [1], [4]. The native Eurasian wild boar (*Sus scrofa*) is regarded as one of the main wildlife reservoirs of MTBC in the Iberian Peninsula [5]–[6]. Moreover, wild boar TB is increasingly recorded in other parts of its geographical range [7]–[8], and feral pig TB is also a concern in countries such as New Zealand and USA [9]. Complete depopulation is normally not an option in the native range of this species, and culling has only transient effects on infection prevalence (unpublished results). Effective wild boar proof fencing of livestock pastures is costly and might cause conflicts with conservationists due to the barrier effect [10]. Hence, wildlife vaccination to reduce MTBC infection prevalence could become a valuable alternative or complementary tool in TB control [11].

Wildlife vaccination for TB control is being studied worldwide in several host reservoir models, including the brushtail possum (*Trichosurus vulpecula*), ferret (*Mustela furo*) and red deer (*Cervus elaphus*) in New Zealand [12]–[14], White-tailed deer (*Odocoileus virginianus*) in the USA [15], African buffalo (*Syncerus caffir*) in South Africa [16] and Eurasian wild boar (*Sus scrofa*) in Spain [17], among others. In the United Kingdom and the Republic of Ireland, vaccination of Eurasian badgers (*Meles meles*) is seen today as a key element in a long-term strategy to eradicate the disease from cattle [18]. The vaccine used in all these experiments is BCG (Bacille Calmette–Guérin), an avirulent live strain of *M. bovis*. In wild brushtail possums, BCG was found to prevent infection and had high (69–95%) protective efficacy [14], [19]. Badgers have also been successfully vaccinated in the field by administering BCG by the intramuscular route. Vaccination reduced the incidence of positive serological test results by 74% [20]. In African buffaloes, oral BCG produced 33% reduction in lesion scores in a study carried out in an open-air enclosure [16]. The oral delivery of baits containing BCG to wild boar in experimental conditions allowed reduction of *M. bovis* infection by 50% and lesion scores by 56% after challenge with *M. bovis* [17]. At the molecular level, oral BCG immunization of wild boar resulted in the upregulation of immunoregulatory genes such as interferon gamma (IFN-gamma), regulated on activation, normal T expressed and secreted cytokine, also known as CCL5 (RANTES), methylmalonyl coenzyme A mutase (MUT), complement component 3 (C3) and interleukin 4 (IL-4) that may be associated with protective response to *M. bovis* infection in this species [17], [21]–[23].

Vaccination against TB has been studied in human tuberculosis and in several animal models with live mycobacteria such as BCG, BCG recombinants and other mutant strains, DNA or protein subunit vaccines, and inactivated (heat- or formalin-killed) vaccines [24]–[27]. Several organisms including *M. bovis* BCG, the leprosy vaccine *Mycobacterium w*, and *Mycobacterium vaccae* have also been used in the form of inactivated vaccines [28]–[32]. In guinea pigs, formalin-inactivated BCG mixed with non-phospholipid liposome adjuvants and administered as a single subcutaneous inoculation conferred a significant survival advantage against lethal aerogenic challenge with *M. bovis* [28]. In cattle however, killed BCG in a mineral-oil adjuvant did not evoke protective immunity [12]. In possums it has been tested if oral killed BCG would adversely affect subsequent vaccination with live BCG, finding that protection was not adversely affected compared to live BCG administered orally [29].

Regarding *M. w*, significantly higher IFN-gamma production was observed in mice immunized with heat-killed organisms by the subcutaneous route than in unvaccinated controls [30]. However, both IFN-gamma production and protection levels were consistently better in live *M. w* vaccinated mice than in those vaccinated with heat-killed *M. w* [30]. A vaccination study with heat-killed *M. w* in India provided evidence suggesting protective efficacy of *M. w* against pulmonary TB in humans [31]. Preventive immunization with whole inactivated *M. vaccae* conferred protection against HIV-associated TB in BCG-immunized human adults [32]. However, intradermal vaccination with 10^9^ heat-killed *M. vaccae* did not protect cattle against an experimental challenge with *M. bovis* and induced only weak cell-mediated immune responses to bovine PPD [33]. Heat-inactivated killed *M. vaccae* administered intranasally/intraconjunctivally to possums induced minimal protection compared to the combination of killed *M. vaccae* and live BCG by the same route [34]. However, to the best of our knowledge there is no peer-reviewed information regarding the single use of oral inactivated vaccines for controlling TB in wildlife. These vaccine formulations for TB control in wildlife would have the advantage of being environmentally safe and more stable under field conditions when compared to live BCG vaccines.

In this study, we hypothesized that wild boar orally and parenterally immunized with inactivated *M. bovis* will produce an antibody response similar to oral live BCG vaccination and natural *M. bovis* infection, but that protection against a challenge with an *M. bovis* field strain, as well as the gene expression and IFN-gamma response would be different using inactivated *M. bovis* and BCG. To test this hypothesis, four groups of 5 wild boar each were vaccinated with inactivated *M. bovis* by the oral and intramuscular routes, vaccinated with oral BCG, or left unvaccinated as controls. All groups were later challenged with a field strain of *M. bovis*. The results of the IFN-gamma response, serum antibody levels, *M. bovis* culture, TB lesion scores, and the expression of C3 and MUT genes were compared between these four groups.

## Materials and Methods

### 1. Animals and experimental design

Twenty 3-4-month-old wild boar piglets were bought in a commercial farm known to be free of mycobacterial lesions at slaughter and with a fully negative ELISA test [35]. The animals were housed in class III bio-containment facilities where they had ad libitum food and water. Wild boar piglets were randomly assigned to one of four treatment groups: Group 1, unvaccinated controls; Group 2, parenterally vaccinated with heat-inactivated *M. bovis*; Group 3, orally vaccinated with heat-inactivated *M. bovis*; Group 4, orally vaccinated with live BCG. Oral vaccines were delivered in baits designed for wild boar piglets [36]. For the challenge, 5 ml of a suspension containing 10^6^ colony forming units (cfu) of an *M. bovis* field strain were administered by the oropharyngeal route as described in previous experiments [17], [37].

The animals were handled nine times during the experiment, including the vaccination (T1, day 1), the challenge two months after vaccination (T2, day 60), and the necropsy four months after challenge and six months after vaccination (T3, day 189). In addition to T1, T2 and T3, blood samples were taken at days 8, 21, and 49 post-vaccination (p.v), and after challenge at days 74, 83, 104 and 133 p.v.

Handling procedures and sampling frequency were designed to reduce stress and health risks for subjects, according to European (86/609) and Spanish laws (R.D. 223/1988, R.D. 1021/2005). The protocol was approved by the Committee on the Ethics of Animal Experiments of the Regional Agriculture Authority (Diputación Foral de Vizcaya, Permit Number: 2731-2009).

### 2. Preparation of inactivated vaccines

The *M. bovis* strain used was a first passage level culture isolated from a naturally infected wild boar in Coletsos medium. The isolate was propagated in Middlebrook 7H9 broth enriched with OADC for 2–3 weeks. Cells were harvested by centrifugation at 2500 x g for 20 minutes and washed twice in PBS. The bacterial pellet was re-suspended in PBS and declumped using a fine needle syringe. The turbidity of this suspension was adjusted to an optical density of 1 McFarland unit. Before inactivation, tenfold serial dilutions were prepared and plated in agar-solidified 7H9 with OADC in quadruplicate to assess the number of cfu in the inoculum. The inoculum was then inactivated in a water bath at 80°C for 30 minutes. Animals in “parenteral inactivated vaccine” and “oral inactivated vaccine” groups were administered with approximately 6×10^6^ bacteria according to cfu counts. The parenteral vaccine (1 ml) was prepared using Montanide ISA 50 V, an oily adjuvant of mannide oleate and mineral oil (Seppic, Castres, France). The oral vaccine consisted of 2 ml of PBS containing the inactivated mycobacteria. This inactivated vaccine was again cultured in duplicate to assure absence of viable *M. bovis*.

### 3. BCG vaccine

The *M. bovis* BCG Danish reference strain (CCUG 27863) was kindly provided by C. Martín (Universidad de Zaragoza, Spain). It was cultured on Coletsos medium (Biomerieux, France), an egg yolk medium without glycerol, and colonies from the slant were scraped and transferred to a sterile tube containing 8–10 glass beads. The suspension was mixed in a vortex for a few seconds and sterile distilled water was added. Then it was allowed to settle for 5 min. The supernatant was adjusted with water to turbidity equal to 1.0 McFarland standard. To calculate the amount of BCG cfu per bait, dilutions from 10−1 to 10−6 were prepared and 0.150 ml from each dilution were cultured in Coletsos medium in duplicate. The tubes were cultured at 37°C and cfu readings were taken after 5 weeks. Each bait contained 0.150 ml of the 1.0 McFarland standard.

### 4. Necropsy, sample collection and histopathology

Wild boar were anesthetized by intramuscular injection of zoletil, and euthanized by captive bolt. A thorough postmortem examination was done to detect the presence of macroscopic lesions. Samples for culture were immediately processed and copies frozen at −80°C for mRNA isolation. After taking out pieces for mRNA, all main lymph nodes (LNs) and the tonsils were serially sliced into 1–2 mm thick slices and carefully inspected for visible TB-compatible lesions. Visceral organs were also carefully inspected, and each lung lobe was considered separately. These TB-compatible lesions were classified based on lesion distribution and lesion intensity, and scored as previously described [17].

Samples of individual tissues were fixed in 10% buffered formalin, embedded in paraffin, sectioned at 4 µm, and stained with hematoxylin–eosin by use of standard procedures. An additional section of those tissues with lesions indicative of tuberculosis was stained by Ziehl–Neelsen (ZN) procedure to detect the presence of acid-fast organisms (AFO).

### 5. Microbiology

The tissues collected were as follows: head lymphoid tissues including the oropharyngeal tonsil and both mandibular, parotid, and retropharyngeal lymph nodes (LNs); lung (each lobe), tracheobronchial LNs and mediastinal LN; spleen, ileocaecal valve, and mesenteric and hepatic LNs. When suspicious lesions were observed in liver, kidney and LNs from other locations, samples from these tissues were also cultured. Samples were thoroughly homogenized in sterile distilled water (2 g in 10 ml or equivalently). Five ml of this suspension was decontaminated and processed following the instructions of the manufacturer to inoculate BBL MGIT tubes supplemented with BBL MGIT PANTA and BACTEC MGIT growth supplement (Becton Dickinson). BBL tubes were incubated for 42 days in a BACTEC MGIT 960 System. The remaining 5 ml were decontaminated in hexadecyl-pyridinium chloride at a final concentration of 0.75% (w/v) for 12–18 h. Samples were centrifuged at 2500 x g for 5 min and pellets cultured in Coletsos tubes (bioMèrieux) at 37°C for 4 months. All isolates were spoligotyped in order to confirm the strain [38].

We defined a culture score for *M. bovis* infection in wild boar, as the number of lymph node or organ samples yielding a *M. bovis* isolate, of the total number of culture attempts (N≥17 samples cultured per wild boar; score range 0–17). Infection level of samples was categorized according to the number of colonies per tube as follows: less than 10 colonies, between 10 and 50 colonies and more than 50 colonies.

### 6. bPPD ELISA test

Serum samples were tested for anti-PPD immunoglobulin G (IgG) antibodies by means of an in-house ELISA using bovine tuberculin purified protein derivative (bovine PPD; CZ Veterinaria SL, Porriño, Lugo, Spain) as antigen and protein G horseradish peroxidase (Sigma-Aldrich Química SA, Madrid, Spain) as a conjugate applying the protocol described by Boadella et al. [35]. Sample results were expressed as an ELISA percentage (E%) that was calculated using the formula [Sample E%  =  (sample OD/2 × mean negative control OD) ×100]. Samples with E%>100 were considered positive.

### 7. Dual-path platform (DPP) TB test

The DPP technology was developed by Chembio Diagnostic Systems, Inc. using selected *M. bovis* antigens. Serum samples were tested as previously described [39] and results were read 20 minutes after adding sample buffer. The presence and intensity of either of the 2 separate test lines (T1, MPB83 antigen; T2, CFP10/ESAT-6 fusion protein) were evaluated by a DPP optical reader (in relative light units, RLU) [35].

### 8. Interferon gamma test

Blood samples were collected into tubes with lithium heparin and shipped to the laboratory at room temperature. Stimulation of whole blood with PBS (nil control), and the avian and bovine purified protein derivative (PPD; CZ Veterinaria, Porriño, Spain) was performed within 8 h of collection as described for other species [40]–[42]. Detection of IFN-gamma in the supernatant was performed using a quantitative ELISA (Pierce Endogen, Rockford, IL, USA) following manufacturer's recommendations.

### 9. Gene expression analysis by real-time RT-PCR

The immunoregulatory genes, complement component 3 (C3) and methylmalonyl-CoA mutase (MUT) were selected for analysis based on their association with wild boar resistance to natural *M. bovis* infection and protection against *M. bovis* challenge in BCG-vaccinated wild boar [6], [17], [21]–[22].

Total RNA was extracted from peripheral blood mononuclear cells (PBMC) using TriReagent (Sigma, Madrid, Spain) following manufacturer's recommendations. The RNA yield and quality were assessed using the Experion Bioanalyzer (Bio-Rad, Madrid, Spain). Gene-specific oligonucleotide primers were designed and used for quantitative real-time RT-PCR (qRT-PCR) [17]. The qRT-PCR was performed in 25 µl reaction volumes with 12.5 µl SYBR Green iScript® (Bio-Rad). Amplification conditions consisted of 95°C for 1 min, followed by 40 cycles of 95°C for 15 s and 55°C for 60 s. A dissociation curve was run at the end of the reaction to ensure that only one amplicon was formed and that the amplicon denatured consistently in the same temperature range for every sample [43]. All reactions were performed in triplicate. Oligonucleotide primers were used to amplify the *S. scrofa* cyclophilin (Genbank accession number AY008846) as a control gene to normalize expression data [21]. Control reactions were performed using the same procedures, but without RT to monitor DNA contamination in the RNA preparations and without RNA added to monitor contamination of the PCR reaction. The mRNA values were normalized against *S. scrofa* cyclophilin gene expression using the 2-ΔΔCt method [44]. The normalized expression was calculated at each time point and the mean of triplicate values was used to compare data between vaccinated and control groups.

### 10. Statistical analyses

Normal data such as body weight, head and body length, and kidney fat were compared by analysis of variance (ANOVA) followed by a series of Tukeýs post-hoc tests for pair comparisons (p = 0.05). Regression was used to analyze the relation between lesion scores and serum antibody levels. Chi square tests were used to compare proportions. Lesion scores and culture scores were compared among groups with the non-parametric median test.

## Results

### 1. Clinical signs and body condition

All animals were observed daily throughout the experiment and TB signs were not detected. At necropsy, all wild boar were measured, weighted, and the kidney fat index (KFI) calculated. Control wild boar consistently had the lowest values, and the difference among groups was significant for weight and for head and body length, but not for the KFI (Table 1).

**Table 1:** Body weight, head and body length, and kidney fat index (KFI) at necropsy for wild boar belonging to four experimental groups (mean ± SE).

Group	Body weight (kg)	Body length (cm)	KFI (%)
Control	18.42±2.5	88.86±2.6	19.52±7.4
Parenteral inactivated	25.67±2.5	102.54±2.6	25.41±6.6
Oral inactivated	28.04±2.3	103.50±2.9	31.76±6.6
Oral BCG	20.66±2.3	93.58±2.6	29.91±6.6

Differences among groups were significant for weight (F_3, 14_ = 3.4, p<0.05) and for head and body length (F_3, 15_ = 6.9, p<0.01), but not for the KFI (F_3, 15_ = 0.6, p>0.05). Post hoc Tukey tests revealed significant differences only for head and body length between the control group and the two inactivated vaccinated groups (p = 0.01).

### 2. Pathology

**Figure 1:**
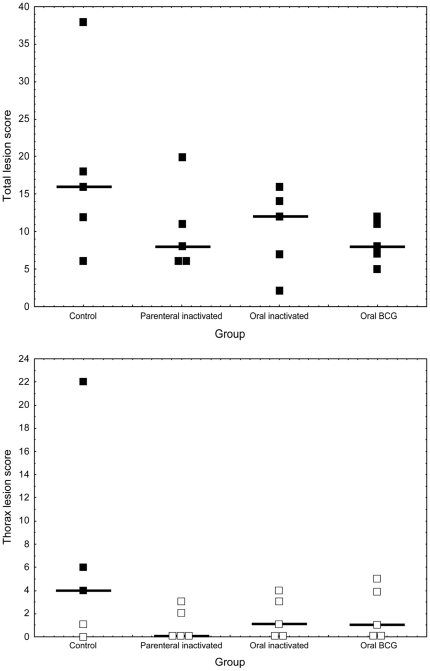
Wild boar TB lesion scores at necropsy. Total lesion score (upper panel) and thorax lesion score (lower panel) for 20 wild boar used in the vaccination and challenge experiments. The solid lines show the median values. In the lower panel, solid squares indicate animals with massive *Mycobacterium bovis* growth (>50 colonies) from thoracic samples.

Total lesion, head lesion and thorax lesion scores were highest in the control group (Figure 1). The mean lesion score for control wild boar was 18±3. In the parenteral inactivated, oral inactivated and oral BCG groups we observed a reduction of the mean lesion score by 43.3%, 43.3% and 52.2%, respectively. One control wild boar presented the most severe lung lesions showing extensive areas of pneumonia involving the cranial, middle and right caudal and left middle lobes, along with both tracheobronchial LNs (Table 2). The reduction of the mean thorax lesion score as compared to the controls was of 84.8%, 75.7% and 69.7%, respectively.

**Table 2:** Summary of pathology findings expressed as number of animals showing macroscopic lesions, number of animals showing only microscopic lesions, and number of animals with AFO in Ziehl-Neelsen stained sections, separated by bars.

	Group			
Organ	Control	Parenteral inactivated	Oral inactivated	Oral BCG
Tonsil	1/0/0	2/1/1	3/0/0	0/1/0
Mandibular LN^a^	5/0/5	5/0/3	5/0/2	5/0/2
Retropharyngeal LN	3/0/0	3/0/2	2/0/1	3/1/0
Parotid LN	1/1/0	2/0/0	1/0/1	0/1/0
Lung	2/0/1	0/1/0	2/0/1	1/1/0
Tracheobronchial LN	4/1/2	3/1/0	2/1/0	3/0/0
Mediastinal LN	1/0/0	0/0/nd	1/0/0	0/0/nd
Liver	0/0/nd	0/0/nd	0/0/nd	1/0/0
Spleen	0/0/nd	0/0/nd	0/0/nd	0/0/nd
Kidney	0/0/nd	0/0/nd	0/0/nd	0/0/nd
Ileocecal valve	0/1/0	0/0/nd	0/0/nd	0/0/nd
Hepatic LN	0/0/nd	0/0/nd	0/0/nd	1/0/0
Ileocecal LN	0/0/nd	2/0/1	0/0/nd	0/0/nd
Mesenteric LN	0/0/nd	2/0/0	0/0/nd	2/0/0

a LN: lymph node;

b nd: not done.

Two controls and one oral inactivated vaccinated wild boar had AFO in lung tissues. These were not recorded in the parenteral inactivated and BCG vaccinated wild boar. Differences in total and thorax lesion scores were not significant (Median test, Chi^2^ ≤5.45; p>0.05).

### 3. *M. bovis* isolation

Total culture scores and thorax culture scores were again highest in the control group (4.4±0.7 and 0.6±0.2, respectively). All isolates belonged to the field *M. bovis* strain used for challenge. In the parenteral inactivated, oral inactivated and oral BCG groups we observed almost no reduction of the mean culture score: 4.5%, 9% and 9%, respectively. However, the reduction of the mean thorax culture score as compared to the controls was of 66.7%, 33.3% and 66.7%, respectively. Moreover, massive *M. bovis* growth on solid media (>50 colonies) was observed among 3/5 controls, 2/5 parenterally vaccinated, and none of 5 oral inactivated and 5 oral BCG vaccinated wild boar, respectively. Tissues where massive growth occurred included thoracic LNs in the three controls (tracheobronchial LN in two cases and mediastinic LN in one), but only head LNs (mandibular and retropharyngeal) in the parenterally vaccinated ones (Figure 1). Differences in total and thorax culture scores were not significant (Median test, Chi^2^ ≤2.42; p>0.05).

### 4. Serum antibody response

**Figure 2:**
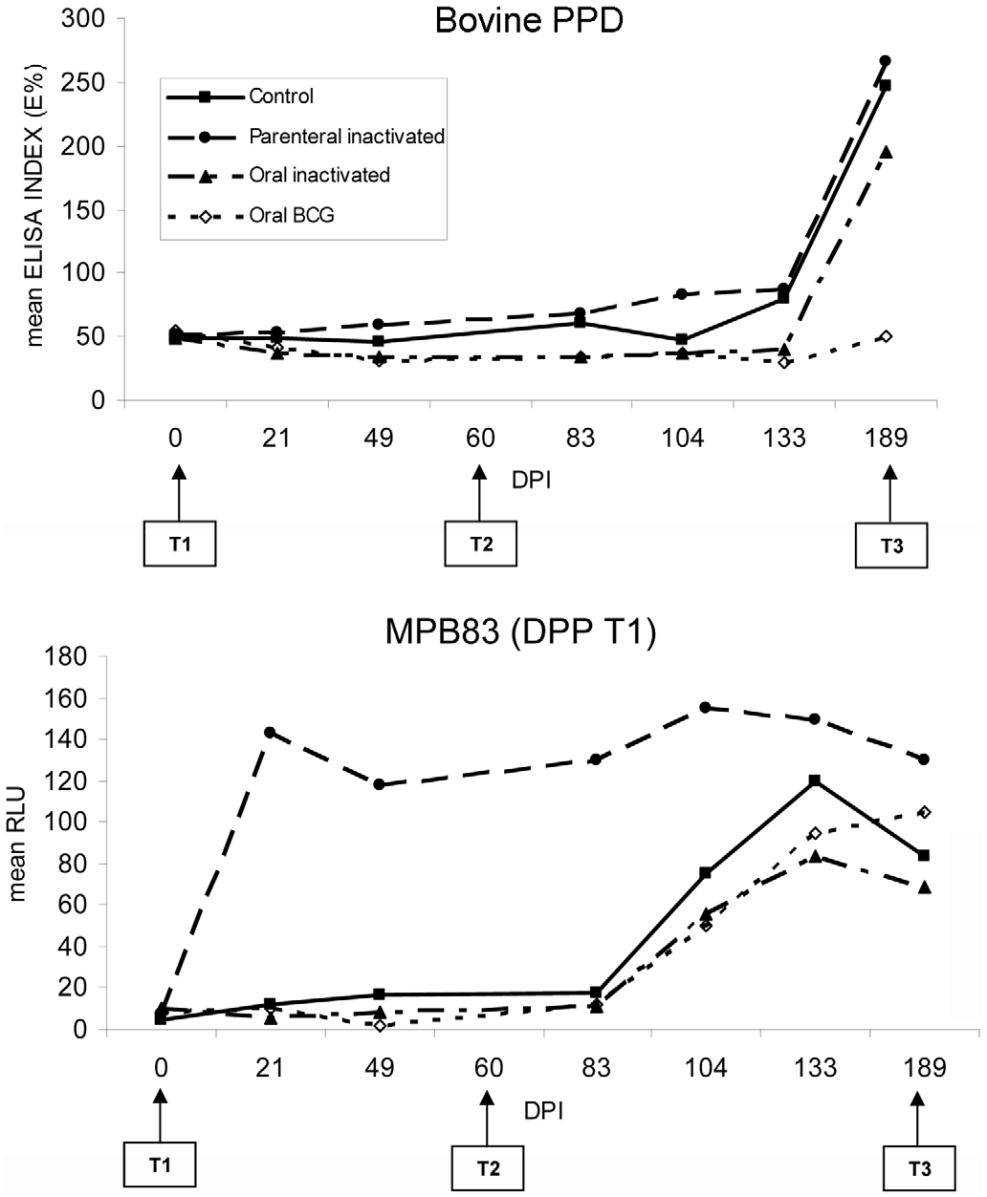
Serum antibody response after vaccination and after challenge. Mean serum antibody response (n = 5 for each point and group) as measured by the bPPD ELISA (in ELISA percentage, E%) and the MPB83 dual-path platform test (DPP-T1, in relative light units, RLU).

Figure 2 presents the mean antibody responses per treatment group. Regarding bPPD, antibody levels were consistently low during the experiment and increased only late after challenge in all groups except the oral BCG one (time F_6, 112_ = 6.92, p<0.001; group F_3, 112_ = 2.62, p>0.05; interaction F_18, 112_ = 0.72, p>0.05). DPP-T1 reactivity changed significantly in time (F_6, 112_ = 23.7, p<0.001) and among groups (F_3, 112_ = 45.2, p<0.001), and the interaction between time and group was also significant (F_3, 112_ = 2.62, p<0.05), essentially because the parenterally inactivated vaccinated wild boar responded to MPB83 antigen immediately after vaccination, in contrast to the orally vaccinated ones and the controls. No significant differences were observed regarding antibody reactivity with CFP10/ESAT-6 in the DPP assay (F<2, p>0.05 in all cases). Thus, the serologic tests allowed differentiating vaccinated not challenged from vaccinated challenged wild boar after a period of time post inoculation, if the vaccine was administered parenterally. The former responded to MPB83 and not to bPPD, while the latter (after challenge) responded to both bPPD and MPB83.

At necropsy, the differences among groups in serum antibody levels were not significant (bPPD: F_3, 16_ = 0.87; DPP-T1: F_3, 16_ = 1.68; DPP-T2: F_3, 16_ = 1.37; p>0.05). However, the oral BCG group tended to have the lowest responses to bPPD and to CFP10/ESTA-6 antigen, while the parenteral inactivated vaccine group had the highest responses to bPPD and to MPB83 protein. Control wild boar had the highest response to CFP10/ESAT-6 antigen in DPP assay (Table 3).

**Table 3:** Serum antibody levels at necropsy for 20 wild boar belonging to four experimental groups (mean ± SE), as measured by the bPPD ELISA (in ELISA percentage, E%) and the dual-path platform tests (DPP, in relative light units, RLU; DPP test 1: MPB83; DPP test 2: CFP10/ESAT-6).

Group	bPPD ELISA %E (+/−)	Chembio DPP test 1 RLU (+/−)	Chembio DPP test 2 RLU (+/−)
Control	247.02±104 (3/2)	83.36±20.7 (4/1)	31.68±11.9 (2/3)
Parenteral inactivated	265.31±104 (4/1)	129.74±20.7 (5/0)	11.80±11.9 (1/4)
Oral inactivated	195.24±104 (2/3)	68.22±20.7 (4/1)	4.7±11.9 (2/3)
Oral BCG	50.25±104 (0/5)	105.28±20.7 (5/0)	0±11.9 (0/5)
Chi^2^ 3 d.f.	7.07 p>0.05	2.22 p>0.05	7.2 p>0.05

**Figure 3:**
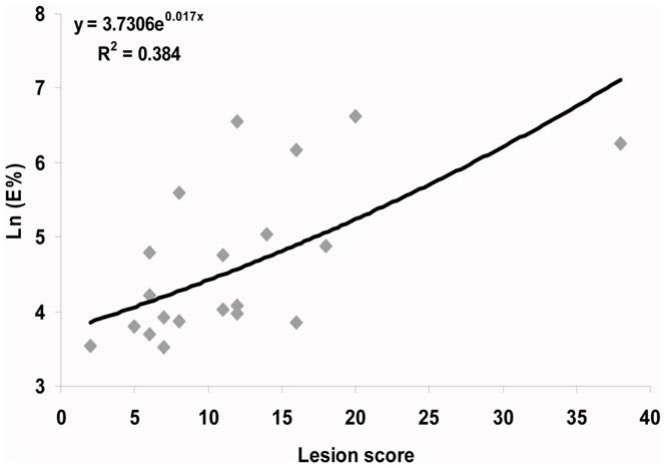
Relationship between antibody response and lesion scores. Relationship between the level of antibodies against bPPD in blood serum (E%) and the lesion scores of wild boar (n = 20) experimentally challenged with *Mycobacterium bovis*. The regression equation and the coefficient of determination are shown in the chart.

We observed a statistically significant positive relationship between the bPPD ELISA values and total lesion scores of the 20 challenged wild boar (R^2^ = 0.38, p<0.01; Figure 3).

### 5. Interferon gamma response

**Figure 4:**
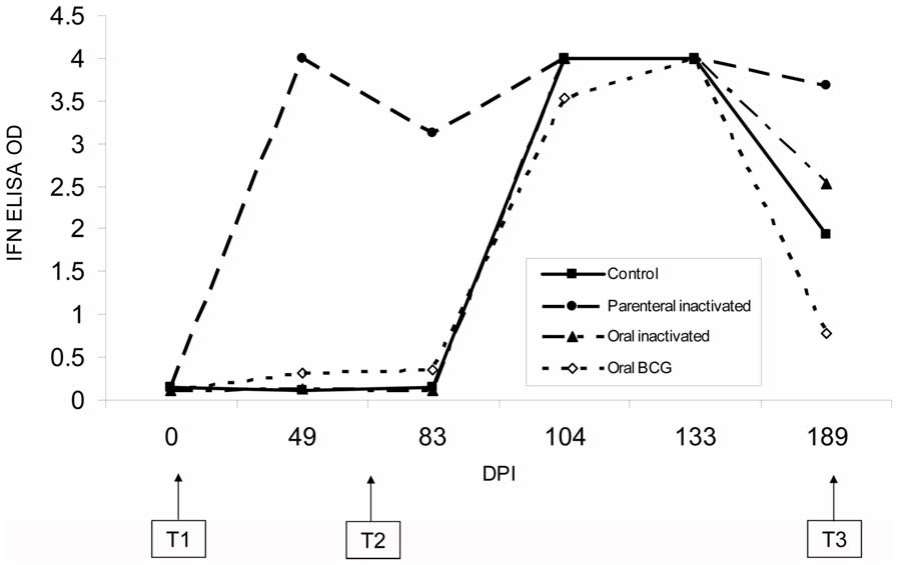
Detection of gamma interferon. Mean optical density (OD) readings of the gamma interferon ELISA (n = 5 for each point and group).

Figure 4 presents the mean OD readings of the four treatment groups regarding IFN-gamma production in response to bPPD. All tested negative to IFN-gamma at T1, mean levels being nil (mean OD 0.131). All PBS controls also yielded consistently low results (OD<0.227). Forty-eight days after vaccination, only the parenterally vaccinated group showed a clear and homogeneous IFN-gamma response. Two wild boar of the oral BCG vaccinated group also responded to a lesser extent. One week after challenge, the picture remained as after vaccination. However, at day 21 after challenge all wild boar showed high IFN-gamma responses which were maintained until necropsy at T3 (OD>0.291). Again, the mean response stayed highest in the parenterally vaccinated group (F_3,16_ = 3.88, p<0.05). Post hoc Tukey tests revealed significant differences only between parenterally vaccinated and oral BCG vaccinated wild boar (p = 0.02). The IFN-gamma OD at T3 was correlated with the number of ZN positive tissues (r_s_ = 0.44, n = 20, p<0.05). We found no other correlation between IFN-gamma responses and culture or lesion scores (p>0.05).

### 6. Gene expression response to vaccination

**Figure 5:**
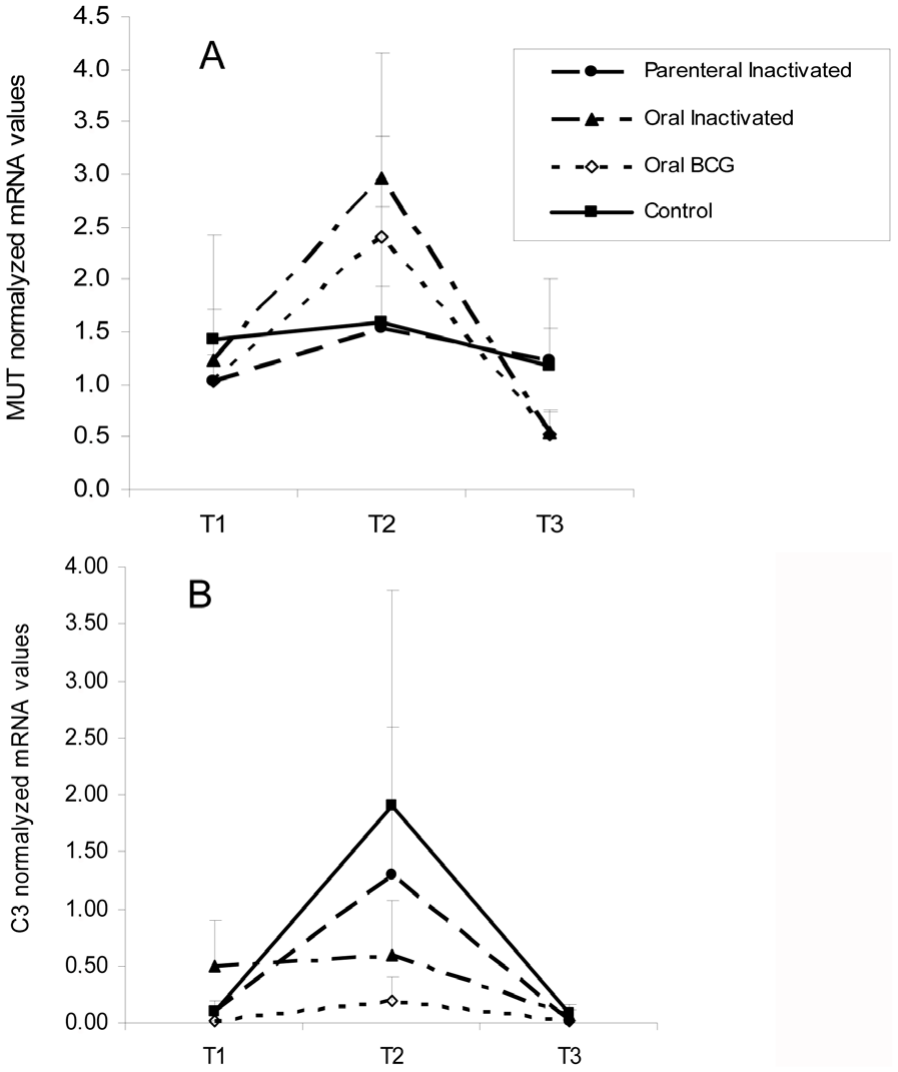
Quantitative MUT and C3 gene expression analysis in PMBC using qRT-PCR. Quantitative MUT (panel A) and C3 (panel B) gene expression analysis in PBMC of vaccinated wild boar and controls using qRT-PCR. Results were recorded as mRNA levels after normalization for cyclophilin gene expression using the 2-ΔΔCt method for each of the three time points (T1: before vaccination, T2: before challenge, T3: end of the experiment). Values are shown as average ±SD.

The C3 and MUT mRNA levels were analyzed in PBMC at time points T1 (before vaccination), T2 (2 months after vaccination and before challenge) and T3 (at the end of the experiment 4 months after challenge; Figures 5A and 5B). MUT expression was similar between wild boar orally-immunized with BCG and inactivated *M. bovis* and was significantly higher (p<0.05) when compared to controls at T2 (Figure 5A). For these groups, MUT expression levels were significantly (p<0.05) higher at T2 than at T1 and T3 (Figure 5A). MUT expression levels in wild boar parentally-immunized with inactivated *M. bovis* were similar to controls at all time points (Figure 5A). Although the C3 expression levels were significantly (p<0.05) higher at T2 than at T1 and T3 for wild boar orally-immunized with BCG, differences were not observed between groups at any time point analyzed.

## Discussion

The number of wild boar tested was necessarily low, and this limited the statistical significance of the results. However, data presented herein suggest that vaccines based on heat-inactivated *M. bovis* triggered an immune response in wild boar that conferred protection against challenge with an *M. bovis* field strain at a similar level as did oral BCG. In contrast to our initial hypothesis, this is the first report showing the potential use of inactivated *M. bovis* vaccines for TB control in a wildlife species.

In order to obtain uniform results, we used a high challenge dose of 10^6^ cfu of *M. bovis* [17]. As a consequence, all vaccinated wild boar subjected to experimental challenge became infected. However, vaccination reduced the number and severity of lesions and the infection burden, particularly in the thoracic region, similar to BCG vaccination results in other wildlife species (e.g. in badgers challenged with 10^4^ *M. bovis* CFU by the endobronchial route; [18], [20]). Thus, given the severity of the challenge, the estimated protection is likely to be an underestimate of the true vaccine effect [18].

Protection levels, antibody levels, IFN-gamma response and MUT and C3 gene expression varied among treatments. Unvaccinated controls responded after challenge with both antibody and IFN-gamma production. At the time of necropsy, controls had the poorest condition scores and the highest mean lesion and culture scores, more thoracic tissues with AFO, as well as the highest mean antibody response to the CFP10/ESAT-6 fusion protein. The lesions recorded in this control group were similar to those observed in previous experiments in captivity [17], and to those recorded in the field [45]. The finding of lower condition scores in controls as compared to vaccinated animals contrasts with results obtained in badgers [18]. However, badgers used for the vaccination and challenge experiments were field-captured adults while we used more uniform farm-bred wild boar piglets.

It is remarkable that all wild boar responded to MPB83 (DPP-T1) immediately after challenge and before antibodies against bPPD raised in the ELISA test. Both techniques can potentially be complementary to differentiate animals with recent infection (and probably without visible lesions) from animals with more advanced lesions. Whether the MPB83 increase is transient or not needs to be investigated.

In contrast to all other treatments, the group vaccinated parenterally with inactivated *M. bovis* had an almost immediate MPB83 and IFN-gamma response after vaccination. This group maintained the highest IFN-gamma levels at necropsy. In badgers, most individuals were also positive to IFN-gamma after parenteral BCG vaccination [20] and in wild boar a transient increase in IFN-gamma mRNA and serum levels were observed 5 weeks after parenteral BCG vaccination [21]. The early and robust IFN-gamma and MPB83 serum antibody responses, in contrast to the less prominent responses to the bPPD ELISA test, would allow using a combination of these tests to distinguish between parenterally vaccinated and parenterally vaccinated/*M. bovis*-infected wild boar in the field, at least until 4 months after challenge. In vaccinated mice and possums, IFN-gamma is considered a correlate of protective immunity [30], [46]. However, in this study we observed no correlation between IFN-gamma response and lesion scores, except for the number of ZN positive tissues. This lack of correlation between post vaccination cellular immune responses and levels of protection was also observed in orally BCG vaccinated badgers, but was not associated with a failure of the vaccine to protect against *M. bovis* [18].

The group vaccinated orally with inactivated *M. bovis* responded in a very similar way to the group vaccinated with oral BCG. Lesion and culture scores were similar, although slightly lower in the BCG group. Regarding serum antibodies and IFN-gamma production, both groups responded only after challenge, as in orally BCG vaccinated badgers [18]. The BCG group had the lowest bPPD and CFP10/ESAT-6 antibody levels, suggesting less advanced disease and/or less stimulation of the antibody response against *M. bovis*. Serum antibodies have been used as a surrogate for BCG-mediated protection against experimental [47] and natural *M. bovis* infection in badgers [20]. Furthermore, our results confirmed that the bPPD ELISA results correlate with the lesion score in wild boar (Figure 3). These results suggest that this ELISA may be used not only for prevalence studies, but also for classifying infected animals as showing a more or less advanced disease, for instance during field experiments to test the effect of vaccination for the control of TB in wild boar.

*M. bovis* infection affects gene expression in wild boar [22]–[23], [48]–[50]. Some of these genes such as C3 and MUT were shown to correlate with resistance to natural *M. bovis* infection and protection against *M. bovis* challenge in BCG-vaccinated wild boar [6], [17], [21]–[22]. As in previous experiments [17], [21]–[23], [50], MUT mRNA levels increased with BCG vaccination and decreased after *M. bovis* infection. However, the increase in MUT expression after vaccination was significant for orally-immunized wild boar only, suggesting differences between orally and parentally immunized animals at least with respect to MUT expression. Although the C3 expression profile in wild boar orally-immunized with BCG was similar to that shown before in parentally and orally BCG-immunized wild boar [17], [21], no significant differences were observed between groups. These results provided additional evidences for the role of MUT expression in protection against *M. bovis* infection in wild boar. Although the mechanism by which MUT expression contributes to resistance to mycobacterial infection are unknown, a hypothesis was recently discussed to suggest that host genetically-defined higher MUT expression levels result in lower serum cholesterol concentration and tissue deposits that increase the protective immune response to *M. bovis*, thus resulting in resistance to bovine TB and better response to BCG vaccination [51]. The observation that both orally vaccinated groups had a clear increase in MUT expression after vaccination suggested that MUT expression, in combination with serum antibody tests, could be used to distinguish between unvaccinated/uninfected, orally vaccinated, and infected or orally vaccinated/infected wild boar in the field. However, a major flaw of using killed *M. bovis* as vaccine would be that (parenteral) vaccination will induce responses to antigens like ESAT6 and CFP-10. These antigens have been suggested as DIVA antigens for BCG vaccination [27]. Hence, wild boar immunization experiments with killed BCG or other *M. bovis* variants should eventually be considered.

### Conclusions

The results reported here showed that wild boar respond similarly to BCG and to vaccination with heat-inactivated *M. bovis*. These results also encourage to test combinations of BCG and inactivated *M. bovis* to vaccinate against TB in wild boar, as tested in possums by Skinner et al. [34] with *M. vaccae*. Vaccine formulations using heat-inactivated *M. bovis* for TB control in wildlife would have the advantage of being environmentally safe and more stable under field conditions as compared to live BCG vaccines. Tools for the selective and effective delivery of baits to wild boar piglets have been set up and field-tested with dummy vaccines [36], [52]–[54]. The ELISA and MUT expression tests shown here can help differentiating between vaccinated and infected animals and as correlates of protective response in vaccinated wild boar. Hence, studies in free-living wildlife under conditions of natural *M. bovis* transmission are now possible and will hopefully reveal the full potential of protecting wild boar against TB using oral *M. bovis* inactivated and BCG vaccines.
